# Metal-Conjugated Affinity Labels: A New Concept to Create Enantioselective Artificial Metalloenzymes

**DOI:** 10.1002/open.201200044

**Published:** 2013-02-20

**Authors:** Thomas Reiner, Dominik Jantke, Alexander N Marziale, Andreas Raba, Jörg Eppinger

**Affiliations:** [a] KAUST Catalysis Center, KCC, King Abdullah University of Science and TechnologyKAUST, Thuwal 23955-6900 (Saudi Arabia); [b] Chemistry Department, Technische Universität MünchenLichtenbergstr. 4, 85748 Garching (Germany)

**Keywords:** affinity labels, artificial metalloproteins, asymmetric catalysis, cysteine proteases, hydrogenation

Dedicated to Professor Wolfgang A. Herrmann on the occasion of his 65^th^ birthday

Incorporation of artificial metal centers into proteins and peptides has emerged as an important tool in chemical and biological research.^1^ Current applications include pharmaceuticals,^2^ probes for molecular imaging^3^ and contrast agents,^4^ tools for biophysical studies targeting metalloprotein functions,^5^ metal-directed protein assembly,^6^ electrochemical biosensors,^7^ and altered electrochemical potential of electron transporting proteins,^8^ as well as the synthesis of functional metalloenzymes with non-natural catalytic activity.^9^ Particularly artificial metalloenzymes received great interest, since they hold the promise to greatly expand the range of reactions accessible by biocatalysis. A variety of methods to generate artificial metal sites were developed including domain-based directed evolution strategies,^10^ engineering of transition-metal binding sites through introduction of coordinating amino acids at geometrically appropriate positions^11^ or site-directed in vivo incorporation of artificial metal-chelating amino acids.^12^ However, the site-directed anchoring of artificial cofactors representing appropriate ligands or metal complexes has so far been the most successful strategy to achieve good catalytic activities and enantioselectivities. Inspired by the pioneering work of Wilson and Whitesides,^13^ Ward revealed the potential of the supramolecular biotin–(strept)avidin technology, which in combination with directed or rationally guided evolution^14^ can deliver highly enantioselective organometallic enzyme hybrid (OMEH) catalysts.^15^ Such biotin–(strept)avidin–metal conjugates were subsequently tested in a variety of catalytic transformations.^16^ In contrast to this supramolecular approach, covalent anchoring of artificial cofactors on proteins can utilize a variety of protein hosts and hence is not limited by the stability range of the biotin–(strept)avidin complex. Originally introduced by Kaiser,^18^ covalent attachment of artificial cofactors has been applied to convert proteases,^19^ lipases^20^ or other non-metal proteins^21^ into organometallic enzyme hybrids. However, none of the covalent approaches could so far achieve the enantioselectivities reached by biotin–(strept)avidin conjugates.9d, 21e Although metals are introduced site-specifically, most systems generated through a covalent approach possess a flexible linker and hence lack a well-defined localization of the metal center on the surface of the host protein, which is a prerequisite to achieve chiral induction.

We reasoned that a well-defined orientation of the metal center in the binding pocket of a protease could be achieved, if the specific binding-affinity pattern of the pocket is utilized to position a suitable artificial cofactor. Correspondingly, we decided to synthesize and test metal-conjugated affinity labels (m-ALs), which consist of 1) an achiral, catalytically active metal complex linked to 2) a protease-specific reactive group able to form a covalent bond with the active center and 3) a peptidic tail to direct the binding orientation of the m-AL (Figure 1). To test the hydrogenation of ketones, we chose a system based on cysteine proteases of the papain family as host proteins. This class of proteases is efficiently inhibited by epoxysuccinyl ester derivatives of the known calpain inhibitor E64c through S-alkylation of the reactive epoxide group by the active site cysteine.^17^, ^22^ Hence, these well-suited, family-wide affinity labels^23^ were chosen to incorporate catalytically active rhodium- or ruthenium-half-sandwich complexes into the enzymatic host. M-ALs were synthesized starting from E64c derivatives (**1**–**3**), which were first converted to the corresponding pentafluorophenyl esters (**1-OPf**–**3-OPf**) and subsequently coupled to monomeric amino-functionalized half-sandwich complexes of ruthenium and rhodium^25^ (**1^Ru^**, **2^Ru^** and **1^Rh^**–**3^Rh^**, respectively; see Scheme 1).

**Figure 1:**
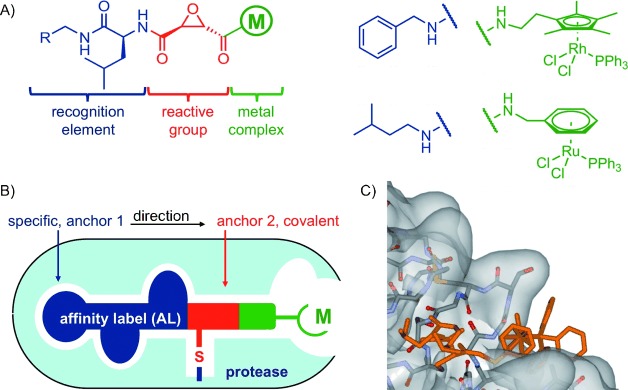
A) Anatomy of metal-conjugated affinity labels (m-ALs) consisting of recognition element, reactive group forming a covalent bond and catalytically active metal complex. B) Concept of utilizing m-AL-directed binding to define the position of the active metal. C) Model of **2^Rh^@papain** based on the X-ray structure of papain with an E64c inhibitor (PDB: 1PPP)^17^ illustrating the predicted position of the metal center in the protease binding pocket.

**Scheme 1:**
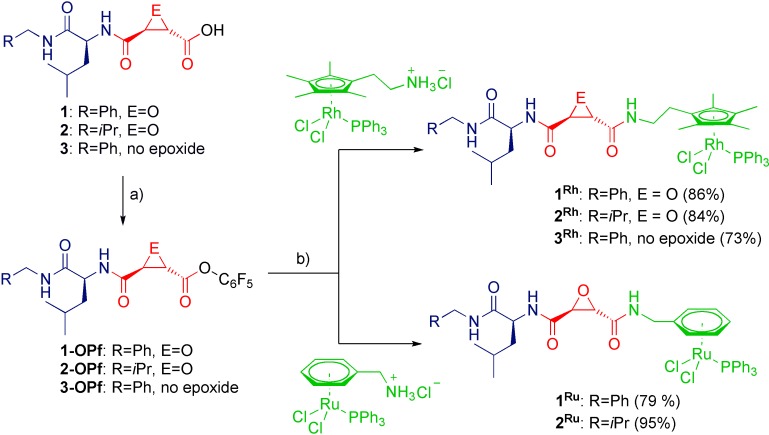
Synthesis of m-ALs used in this study. *Reagents and conditions:* a) pentafluorophenol (1.5 equiv), PS-*N*,*N*′-dicyclohexylcarbodiimide (3 equiv), CH_2_Cl_2_, 0°C→RT, 8 h; b) metal complex (1.0 equiv), Et_3_N (4.5 equiv), CH_2_Cl_2_, RT, 0.5 h.

Cysteine proteases of the papain family are readily converted into organometallic enzyme hybrids through conjugation with m-ALs **1^Rh^, 1^Ru^** or **2^Rh^** (Figure 1 C). Because this system is modular, combination of different affinity labels, metal complexes and proteases allows a straightforward generation of OMEH catalyst libraries, from which promising candidates can be selected based on their performance in asymmetric catalysis. In this study, we generated a variety of OMEH catalysts originating from three affinity labels (**1**, **2** and **3**), two metal complexes (Rh and Ru) and three cysteine proteases (papain, bromelain and cathepsin L). MALDI-TOF MS was used to confirm selective formation of the desired enzyme hybrids. Upon addition of m-AL **1^Rh^** to papain a new signal at 23.976 kDa appeared, indicating formation of the desired organometallic enzyme hybrid (**1^Rh^@papain**, Figure 2). After incubating papain with one equivalent of m-AL **1^Rh^** for two hours the signal of the corresponding OMEH was predominantly observed (papain/**1^Rh^@papain**=1:5). The observed mass increase (Δ*M*=582 Da) compared to free papain (567 Da for addition of **2^Rh^**) indicates loss of the triphenylphosphane and chloride ligands, which may explain the accelerated catalytic rates observed for protease-bound metal centers (see below). To rule out that metal coordination by nucleophilic amino acid side chains is responsible for m-AL binding, we compared the conversion of papain with the metal free m-AL precursors (**1** and **2**)^26^ and m-AL **3**, which is lacking the epoxide moiety. Whereas a selective 1:1 addition was found for the precursors (**1** and **2**), m-AL **3** did not bind to the protein, indicating that the epoxide function is required for covalent OMEH formation. Conversion with the ruthenium-based m-ALs **1^Ru^** and **2^Ru^** also resulted in selective 1:1 addition, yet the observed masses (**1^Ru^**: Δ*M*=420 Da, **2^Ru^**: Δ*M*=401 Da) suggest dissociation of the arene–Ru bond. Because η^*6*^*-*arene–Ru complexes were shown to be stable under similar reaction conditions,19b, 24, 27 and because we observed hydrogenation activity with asymmetric induction for the corresponding OMEHs, we assume that the observed dissociation results from the instability of the arene–Ru moiety under laser irradiation during MALDI-TOF measurements. While such complexes were observed by MALDI-MS, their decomposition in solution and solid state is documented.^28^ Selective binding of m-ALs at the active site of the proteases is further supported through an indirect method based on the decrease of proteolytic activity observed in a chromogenic assay using *N*-benzoyl-dl-arginine *p*-nitroanilide (BAPNA).^29^ After incubation of papain with an m-AL for 45 min, the release rate of *p*-nitroaniline decreases linearly with increasing m-AL concentration and is indistinguishable from the background reaction at protease/m-AL ratios of 1:1 after sufficient incubation time (Figure S-3 in the Supporting Information). Protease activity is completely suppressed at m-AL concentrations above 7.5 μM. When the same experiment was repeated with **3^Rh^**, there was less than 5 % loss in activity detected at the same inhibitor concentration.

**Figure 2:**
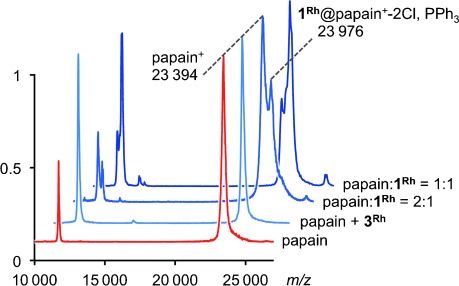
MALDI-TOF mass spectra of papain before and after incubation with m-ALs **1^Rh^** and **3^Rh^**.

Activities and enantioselectivities of OMEHs were determined in the catalytic hydrogenation of ketones. η^*6*^*-*Arene complexes of ruthenium are known to efficiently catalyze this reaction.^30^ 2,2,2-Trifluoroacetophenone served as a model substrate, and conversion was monitored using ^19^F NMR spectrometry. Active OMEH catalysts were prepared by a three-step protocol: 1) activation of the selected cysteine protease using dithiothreitol (DTT) as reducing agent, 2) addition of 0.8 equivalents of m-AL, 3) quenching of residual enzyme activity by addition of **1** in excess to avoid autolytic cleavage of the protease. Substoichiometric addition of the catalyst ensures full conversion of the m-AL to the corresponding OMEH and hence full embedment of the metal centers in the chiral protein ligand.^31^ Following this protocol, m-ALs **1^Rh^**, **1^Ru^** **2^Rh^**, **2^Ru^** and **3^Rh^** were tested for their catalytic performance in combination with papain and bromelain as protein hosts. After in situ addition of the ketone to the freshly prepared OMEH catalyst solution, the reaction was initiated by pressurizing the mixture with hydrogen gas. Comparison of the results achieved for the free m-ALs, the noncovalent m-AL **3^Rh^** in combination with papain, and **1^Rh^@papain** reveals a distinct increase in both activity and enantioselectivity only for a well-defined m-AL–protein interaction (**3^Rh^**≅**1^Rh^**≅**3^Rh^**+papain<**1^Rh^@papain**; see e.r. and yields in Table 1). The protease appears to act as an accelerating asymmetric ligand, a concept which promotes chiral induction.^32^ While this effect is most visible for **1^Rh^@papain** and **1^Ru^@papain**, it is also observed for all other tested OMEH catalysts (Table S-10 in the Supporting Information).

Utilizing the modularity of the system, we were able to identify *R*- and *S*-selective m-AL/protease combinations. Inversion of the stereopreference can result from changes in the metal (62 % *R* for **1^Rh^@papain** versus 60 % *S* for **1^Ru^@papain**; Table 1, Entries 1 and 3), indicating that variations in the metal environment of the OMEH directly affect chiral induction.

**Table 1:** Selected hydrogenation results.[a]

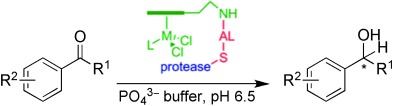							

Entry	Catalyst	*t* [h]	*p* 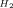 [bar]	R^1^	R^2^	Yield [%][b]	e.r. (*R*/*S*)[c]
1	**1^Rh^@papain**	96	75	H	CF_3_	89	62:38
2	**1^Rh^@papain**	65	25	H	CF_3_	81	62:38
3	**1^Ru^@papain**	96	75	H	CF_3_	44	60:40
4	**1^Ru^@papain**	65	25	H	CF_3_	19	63:37
5	**1^Ru^@bromel.**	96	75	H	CF_3_	44	40:60
6	**1^Ru^@bromel.**	65	25	H	CF_3_	12	61:59
7	**2^Rh^@papain**	65	35	H	CF_3_	41	60:40
8	**3^Rh^**+papain	65	25	H	CF_3_	27	49:51
9	**1^Rh^**	65	25	H	CF_3_	4	52:48
10	**1^Ru^**	65	25	H	CF_3_	5	50:50
11	**3^Rh^**	65	25	H	CF_3_	4	52:48
12	**1^Rh^@papain**	96	75	*o*-F	CH_3_	12	65:35
13	**1^Rh^@papain**	96	75	*p*-CF_3_	CH_3_	16	73:27
14	**1^Rh^@papain**	96	75	*p*-Cl	CH_3_	12	82:18

[a] *Reagents and conditions:* ketone (20 mm), ketone/M (100:1), DTT (0.95 mM), DMSO (12.4 %), phosphate buffer (125 mM, pH 6.5), 40 °C in 525 μL.

[b] Yields were determined using ^19^F NMR spectroscopy.

[c] Enantiomeric ratios were determined using chiral GC.

Screening of reaction parameters revealed that temperature and hydrogen pressure predominantly influence the activity, whereas the pH value, DTT concentration and DMSO content also influence OMEH stereoselectivity. Notably, higher temperature and increased pressure influence kinetics and enhance the hydrogenation rate (Figure 3 A,B). OMEH **1^Rh^@papain** is stable enough to reach constant stereoinduction, and hence conversion of trifluoroacetophenone was nearly quantitative at 40 °C and 75 bar while the enantiomeric ratio remained stable. DMSO content, DTT concentration and the pH value not only influence the hydrogenation reaction but are also critical for the m-AL/protease conjugation, thus, influencing the formation efficiency of the OMEH catalyst. DMSO is required to solubilize the m-AL in the aqueous buffer and higher levels of this cosolvent lead to an increased incorporation efficiency of the metal-conjugated affinity label (Table S-2 in the Supporting Information). DTT is crucial to activate proteolytic cysteine residue of the protease, however, the dithiol can also chelate thiophilic group 8 and group 9 metal centers. Hence, DTT concentrations above 0.95 mM lead to deactivation of the OMEH catalyst, while less DTT results in ineffective covalent bond formation (Figure 3 C). Results without added DTT are similar to those achieved for the noncovalent m-AL **3^Rh^**. A pH screen revealed an optimal pH of 6.5 for **1^Rh^@papain** (Figure 3 D). The shape and maximum of the activity and selectivity curve determined for this OMEH catalyst are similar to that of the bell-shaped activity curve of papain. We reason that the pH value of the reaction not only influences the stability of papain, but also the formation efficiency of the OMEH catalyst and thus determines the ratio of selective and active **1^Rh^@papain** of the total rhodium concentration. Overall, efficient covalent anchoring of the m-AL in the protease binding site is a key factor to achieve good catalytic activity and stereoinduction. The rhodium and ruthenium half-sandwich motives render good conversions only for electron-poor ketones. Stereoinduction is very sensitive to the substitution pattern (Table 1), resulting in major enantiomer contents of up to 82 % (*R*) for 4′-chloroacetophenone. Other covalent papain/Rh or papain/Ru hybrids reported so far achieved only slight enantiomeric enrichment in asymmetric hydrogenations.19d, 24

**Figure 3:**
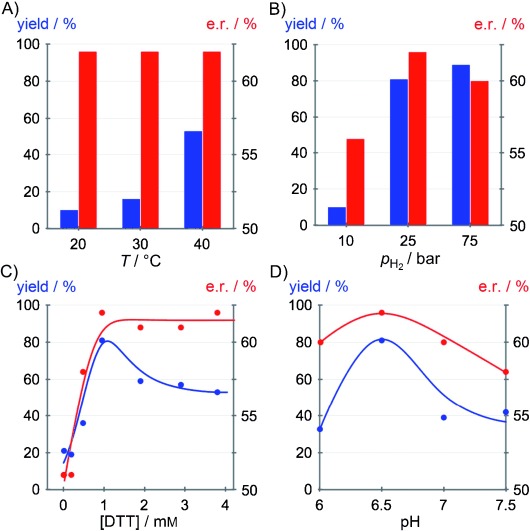
Influence of A) reaction temperature, B) hydrogen pressure, C) DTT concentration and D) pH value applied in the in situ protocol on yields and e.r. values achieved in the OMEH-catalyzed hydrogenation of 2,2,2-trifluoroacetophenone. If not specified otherwise, reaction conditions were as in Table 1, Entry 1. In A), a DTT concentration of 3.8 mM was used.

In conclusion, we have introduced a new platform, which provides rapid access to well-defined and catalytically active artificial metalloenzymes. Our method utilizes specifically designed metal-conjugated affinity labels (m-ALs) to position metal centers inside the binding pockets of proteases. We reason that similar to biogenic cofactors, the affinity tail contributes to the binding process of the m-AL and thus influences the orientation of the metal center relative to the protein surface. In the hydrogenation of ketones, ligand acceleration helps to translate the well-defined metal environment into enantiomeric ratios of up to 82:18. Changing the combination of host protein and metal center alters or even inverts the stereoinduction of the catalyst. The modularity of this platform and the in situ generation protocol should facilitate synthesizing diverse libraries of OMEH catalysts, which can be screened for promising leads for further optimization. The sensitivity of the artificial metalloenzyme characteristics to a change of host protein as well as literature precedence^16^, ^21^ suggest that directed evolution and/or screening of m-AL/protease combinations can be used to improve these OMEH catalysts.

## Supplementary material

As a service to our authors and readers, this journal provides supporting information supplied by the authors. Such materials are peer reviewed and may be re-organized for online delivery, but are not copy-edited or typeset. Technical support issues arising from supporting information (other than missing files) should be addressed to the authors.
