# Supplementary material for: Metal-Conjugated Affinity Labels: A New Concept to Create Enantioselective Artificial Metalloenzymes
Source: ChemistryOpen. 2013 Feb 20;2(2):50–4. doi: 10.1002/open.201200044 (PMC3646430; doi:10.1002/open.201200044)
Supplement: Supplementary file 1 [file open0002-0050-SD1.pdf]

ChemistryOPEN

Supporting Information

© 2013 The Authors. Published by Wiley-VCH Verlag GmbH & Co. KGaA, Weinheim

Metal-Conjugated Affinity Labels: A New Concept to Create Enantioselective Artificial Metalloenzymes

Thomas Reiner,[b] Dominik Jantke,[a] Alexander N. Marziale,[a] Andreas Raba,[b] and Jörg Eppinger*[a]

open_201200044_sm_miscellaneous_information.pdf

Table of contents

**1.**	**Synthetic Methods**		**pg. 2**
	1.1	General remarks	pg. 2
	1.2	Synthesis of non-covalent metalla affinity label precursor **3**	pg. 2
	1.3	General procedure for the synthesis of Rhodium and Ruthenium substituted metalla affinity labels	pg. 3
	1.3.1	Rhodium-substituted metalla affinity label $\mathbf{1^{Rh}}$	pg. 3
	1.3.2	Ruthenium-substituted metalla affinity label $\mathbf{1^{Ru}}$	pg. 4
	1.3.4	Rhodium substituted metalla-affinity label $\mathbf{2^{Rh}}$	pg. 4
	1.3.5	Ruthenium substituted metalla-affinity label $\mathbf{2^{Ru}}$	pg. 5
	1.3.6	Rhodium-substituted non-covalent metalla affinity label $\mathbf{3^{Rh}}$	pg. 5
**2**	**Conjugation of metalla-affinity labels to cystein proteases**		pg. 6
	2.1	Preparation of organometallic enzyme hybrids for MALDI-TOF/TOF analysis	pg. 6
	2.1.1	MALDI TOF/TOF analysis of Papain based organometallic enzyme hybrids	pg. 6
	2.1.2	MALDI TOF/TOF analysis of Cathepsin L based organometallic enzyme hybrids	pg. 6
	2.2	Chromogenic assays for the determination of residual enzyme activity of organometallic enzyme hybrids	pg. 7
**3**	**Hydrogenation of ketones using organometallic enzyme hybrids**		pg. 9
	3.1	General procedure for hydrogenation of ketones	pg. 9
	3.2	Prochiral substrates for asymmetric hydrogenation used in this work	pg. 9
	3.3	Variation of different parameters	pg. 10
**4**	**References**		pg. 12

1 Synthetic methods

1.1 General remarks

Elemental analyses were obtained from the Microanalytical Laboratory of Technische Universität München. IR spectra were recorded on a Jasco FT/IR-460 PLUS (KBR pallets). Mass spectra were recorded on a Thermo Electron LCQ classic. NMR spectra were recorded an a JEOL JNM-GX 400 (^{1}H NMR 400.1 MHz, ^{13}C NMR 100.5 MHz, ^{31}P NMR 161.8 MHz, ^{19}F NMR 376.2 MHz) at T = 300 K, calibration to the residual proton resonance and the natural abundance ^{13}C resonance of the solvent ($CDCl_3$, δ_H = 7.26 and δ_C = 77.00 ppm). Signal multiplicities are abbreviated as: s (singlet), d (doublet), t (triplet), m (multiplet), br (broad). The assignment of proton signals was confirmed by ^{1}H COSY experiments.

All reactions were carried out under an atmosphere of argon using common Schlenk techniques. Dichloromethane and triethylamine were dried over calcium hydride, distilled and degassed prior to use. Polysterene bound cyclohexyl carbodiimide was obtained from Novabiochem. Papain (recrystallized twice) and Bromelain were obtained from Sigma Aldrich or Hoffmann La Roche. Cathepsin L2 was received from Proteros Biostructures, Martinsried. Ruthenium(III) chloride hydrate, Rhodium(III) chloride hydrate and all other reagents were obtained from Sigma Aldrich and used without further purification. 2,5-Dihydro-benzylamine[1], $[(\eta^6\text{-benzylammonium})RuCl_2]_2Cl_2$, $[(\eta^6\text{-benzylammonium})RuCl_2(PPh_3)]Cl$[2], $C_5Me_4H_2(CHCH_2)NH_2$[3], $[(\eta^5\text{-}Me_4Cp(CH_2)_2NH_3)RhCl_2(PPh_3)]Cl$[4], HO-Leu-CONHBn, HO-Epx-Leu-CONHBn (**1-OH**) and HO-Epx-Leu-$CONHCH_2CH_2CH(CH_3)_2$ (**2-OH**)[5] were synthesized according to known literature procedures.

Synthesis of monomeric precursors is required, since the very low solubility of dimeric Rh and Ru complexes in common organic solvents prevents synthesis of m-AL.

1.2 Synthesis of non-covalent metal-conjugated affinity label precursor HO-deoxEpx-Leu-CONHBn **3-OH**

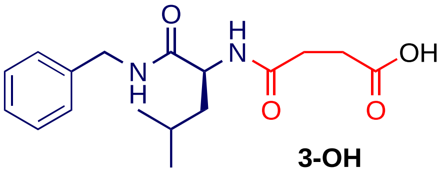

3-OH

Succinic anhydride (245 mg, 2.45 mmol, 1.2 eq) and pyridine (166 µL 2.04 mmol, 1.0 eq) were dissolved in dichloromethane (75 mL) and HO-Leu-NHCOBn (450 mg, 2.04 mmol, 1.0 eq) was added. The reaction mixture was stirred for 24 h at RT. Volatiles were removed under reduced pressure and the crude product was purified via flash chromatography (ethyl acetate / methanol = 15 / 1), yielding a white solid (537 mg, 1.68 mmol, 82 %).

$C_{17}H_{24}N_2O_4$ (320.4): calcd. C 63.73, H 7.55, N 8.74; found C 63.42, H 7.39, N 8.61.

^{1}H-NMR (400 MHz, $CDCl_3$, RT): δ (ppm) = 9.17 (s, 1H, COO*H*), 7.71-7.61 (m, 1H, N*H*CHH’), 7.55 (d, 1H, $^{3}J_{HH}$ = 8.1 Hz, N*H*CH), 7.29-7.11 (m, 5H, $C_{Bn}H$), 4.65-4.52 (m, 1H, NHC*H*), 4.35 (dd, 1H, $^{2}J_{HH}$ = 14.9 Hz, $^{3}J_{HH}$ = 5.8 Hz, NHC*H*H’), 4.19 (dd, 1H, $^{2}J_{HH}$ = 14.9 Hz, $^{3}J_{HH}$ = 5.0 Hz, NHCH*H*’), 2.62-2.28 (m, 4H, CH_2CH_2), 1.68-1.46 (m, 3H, $CH_2CH(CH_3)_2$), 0.88 (d, $^{3}J_{HH}$ = 4.7 Hz, CH_3), 0.85 (d, $^{3}J_{HH}$ = 4.6 Hz, CH’$_3$).

^{13}C-NMR (101 MHz, $CDCl_3$, RT): δ (ppm) = 176.3 (*C*OO), 173.2 / 172.5 (*C*ON), 137.9 (C_{Bn}), 128.6 (C_{Bn}H), 127.6 (C_{Bn}H), 127.5 (C_{Bn}H), 52.0 (*C*HCH$_2$), 43.5 (NH*C*H$_2$), 41.3 (CH*C*H$_2$), 30.6 / 29.6 (*C*H$_2$C’H$_2$ / CH$_2$*C*’H$_2$), 24.9 (*C*H(CH$_3$)$_2$), 22.8 / 22.3 (*C*H$_3$ / *C*’H$_3$).

ESI-MS m/z [%] = 679.3 $[2M+K]^+$ (100), 321.1 $[M+H]^+$ (76), 663.4 $[2M+Na]^+$ (51), 343.3 $[M+Na]^+$ (32).

1.3 General procedure for the synthesis of metal-conjugated affinity labels

Dichloromethane (10 ml) was added to polystyrene bound cyclohexyl carbodiimide (0.24 mmol, 1.3 mmol/g) at 0° C and the resin was allowed to swell for several minutes. The corresponding carboxylic acid (**1** or **2**; 0.08 mmol) was added to the suspension. After 15 min, pentafluorophenol (0.12 mmol) in dichloromethane was added slowly. The resulting suspension was stirred at 0° C for 1 h, warmed to RT and stirred for further 8 h. After filtration, the metal complex (0.08 mmol) and triethylamine (0.36 mmol) were added at RT. After 30 min the reaction mixture was extracted with water and brine (each 3 x 10 ml), dried over $MgSO_4$ and the solvent was removed under reduced pressure. The resulting red solid was washed with diethylether and hexanes (each 3 x 10 ml) to yield the analytically pure metal-conjugated affinity label.

1.3.1 Rhodium-conjugated affinity label $\mathbf{1^{Rh}}$

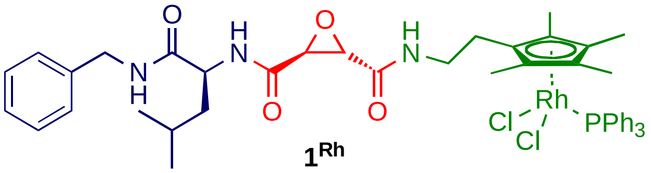

Product: red solid (63 mg, 0.069 mmol, 86 %).

$C_{46}H_{53}Cl_2N_3O_4PRh$ (916.7): calcd. C 60.27, H 5.83, N 4.58; found C 60.22, H 6.09, N 4.34.

^{1}H-NMR (400 MHz, $CDCl_3$, RT): δ (ppm) = 7.82-7.72 (m, 6H, $C_{PPh3}H$), 7.50-7.17 (m, 15H, $C_{PPh3}H$ / $C_{Bn}H$ / CH_2CH_2NH), 7.09-6.95 (m, 2H, N*H*CH$_2$Ph / N*H*CH), 4.48-4.40 (m, 1H, NHC*H*), 4.35 (dd, 1H, $^2J_{HH}$ = 14.9 Hz, $^3J_{HH}$ = 5.8 Hz, NHC*H*H’Ph), 4.27 (dd, 1H, $^3J_{HH}$ = 14.9 Hz, $^2J_{HH}$ = 5.8 Hz, NHCH*H*’Ph), 3.55 (d, 1H, $^3J_{HH}$ = 1.9, C*H*(O)CH’), 3.53 (d, 1H, $^3J_{HH}$ = 1.9, CH(O)C*H*’), 3.38 (m, 2H, CH_2CH_2NH), 2.26 (m, 2H, CH_2CH_2NH), 1.69-1.50 (m, 3H, $CH_2CH(CH_3)_2$), 1.49 (d, 3H, $^4J_{PH}$ = 2.9 Hz, $C_{Cp}CH_3$), 1.48 (d, 3H, $^4J_{PH}$ = 3.1 Hz, $C_{Cp}CH_3$), 1.09 (d, 6H, $^4J_{PH}$ = 2.9 Hz, $C_{Cp}CH_3$), 0.83 (d, 3H, $^3J_{HH}$ = 3.1 Hz, CHC*H*$_3$), 0.81 (d, 3H, $^3J_{HH}$ = 3.1 Hz, CHC*H*’$_3$).

^{13}C-NMR (101 MHz, $CDCl_3$, RT): δ (ppm) = 171.5 / 166.5 / 166.2 (each s, *C*ON), 138.2 (s, C_{Bn}), 134.8 (d, $^3J_{PC}$ = 9.7 Hz, C_{PPh3}), 130.7 (br s, C_{PPh3}), 128.7 (s, C_{Bn}), 128.1 (br s, C_{PPh3}), 127.7 (s, C_{Bn}H), 127.4 (s, C_{Bn}H), 102.5 (d, $^1J_{RhC}$ = 5.5 Hz, C_{Cp}), 102.3 (d, $^2J_{RhC}$ = 4.3 Hz, C_{Cp}), 100.3 (dd, $^2J_{RhC}$ = 6.6 Hz, $^3J_{PC}$ = 6.6 Hz, C_{Cp}), 97.1 (m, C_{Cp}), 54.5 / 54.1 (each s, *C*H(O)C’H / CH(O)*C*’H), 51.8 (s, *C*HCH$_2$), 43.5 (s, NH*C*H$_2$), 41.0 (s, CH*C*H$_2$), 36.3 (d, $^4J_{PC}$ = 4.3 Hz, *C*H$_2$CH$_2$NH), 24.8 (s, *C*H(CH$_3$)$_2$), 24.6 (s, CH$_2$*C*H$_2$NH), 23.0 / 22.1 (each s, *C*H$_3$ / *C*’H$_3$), 9.5 (s, $Cp_{arom}CH_3$), 8.7 (s, $Cp_{arom}CH_3$).

^{31}P-NMR (162 MHz, $CDCl_3$, RT,): δ (ppm) = 30.0 (d, $^1J_{PRh}$ = 143 Hz).

ESI-MS: m/z [%] = 880.1 $[M\text{-}Cl]^+$ (100), 618.2 $[M\text{-}PPh_3\text{-}Cl]^+$ (70).

1.3.2 Ruthenium-conjugated affinity label $\mathbf{1}^{Ru}$

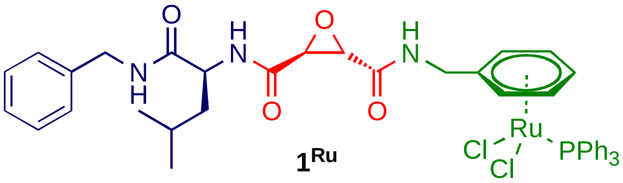

$\mathbf{1}^{Ru}$

Product: orange-red solid (68 mg, 0.079 mmol, 79 %).

$C_{42}H_{44}Cl_2N_3O_4PRu$ (857.77): calcd. C 58.81, H 5.17, N 4.90; found C 58.47, H 5.09, N 4.64.

^{1}H-NMR (400 MHz, $CDCl_3$, RT): δ (ppm) = 7.97 (t, 1H, $^3J_{HH}$ = 5.4 Hz, N*H*CH_2), 7.76-7.65 (m, 6H, C_{PPh3}*H*), 7.51-7.22 (m, 14H, C_{PPh3}*H* / C_{Bn}*H*), 6.76 (d, 1H, $^3J_{HH}$ = 8.3 Hz, N*H*CH), 6.50 (t, 1 H, $^3J_{HH}$ = 5.4 Hz, N*H*CH_2), 5.61 (d, 1H, $^3J_{HH}$ = 5.6 Hz, C_{ortho}*H*), 5.35-5.31 (m, 1H, C_{meta}*H*), 5.29 (d, 1H, $^3J_{HH}$ = 6.1 Hz, C_{ortho}*H*), 5.12-5.04 (m, 1H, C_{meta}*H*), 4.68 (dd, 1H, $^2J_{HH}$ = 15.4 Hz, $^3J_{HH}$ = 6.2 Hz, C*H*H‘), 4.61-4.55 (m, 1H, C_{para}*H*), 4.52-4.30 (m, 5H, PhCH_2 / CH*H*‘ / CH_2Ph), 3.93 (s, 1 H, C*H*(O)CH’), 3.57 (s, 1 H, CH(O)C*H*’), 1.73-1.50 (m, 3 H, CH_2^{i}Pr / CH_2C*H*$(CH_3)_2$), 0.89 (d, 6H, $^3J_{HH}$ = 6.0 Hz).

^{13}C-NMR (101 MHz, $CDCl_3$, RT): δ (ppm) = 171.4 / 167.1 / 166.3 (each s, *C*ON), 138.2 (s, C_{Bn}), 134.2 (d, J_{PC} = 9.2 Hz, C_{PPh3}), 133.0 (d, J_{PC} = 47.7 Hz, C_{PPh3}), 130.8 (d, J_{PC} = 2.5 Hz, C_{PPh3}), 128.7 (s, C_{Bn}H), 128.4 (d, J_{PC} = 10.1 Hz, C_{PPh3}), 127.8 (s, C_{Bn}H), 127.5 (s, C_{Bn}H), 107.6 (d, $^2J_{PC}$ = 6.7 Hz, C_{arom}), 89.5 (s, C_{arom}), 88.0 (d, $^2J_{PC}$ = 6.5 Hz, C_{arom}), 87.6 (s, C_{arom}), 86.2 (s, C_{arom}), 82.0 (s, C_{arom}), 54.54 / 54.46 / 51.9 (each s, *C*H(O)C’H / CH(O)*C*’H / *C*HCH2), 43.5 / 41.0 / 40.6 (each s, NH*C*H_2C_{arom} / NH*C*H_2C_{Bn} / CH*C*H_2), 24.9 / 23.0 / 22.1 (each s, *C*H(CH_3)(C’H_3) / CH(*C*H_3)(C’H_3) / CH(CH_3)(*C’*H_3)).

^{31}P-NMR (162 MHz, $CDCl_3$, RT): δ (ppm) = 27.8 (s).

ESI-MS: m/z [%] = 822.1 $[M\text{-}Cl]^+$ (100), 1679.8 $[2M\text{-}PPh_3\text{-}Cl]^+$ (20), 1415.8 $[2M\text{-}PPh_3\text{-}Cl]^+$ (8).

1.3.3 Rhodium-conjugated affinity label $\mathbf{2}^{Rh}$

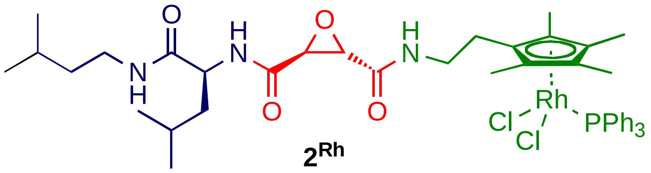

$\mathbf{2}^{Rh}$

$C_{44}H_{57}Cl_2N_3O_4PRh$ (896.73): calcd. C 58.93, H 6.41, N 4.69; found C 58.47, H 5.09, N 4.64.

Product: orange solid (75 mg, 0.84 mmol, 84%)

^{1}H-NMR (400 MHz, $CDCl_3$, RT): δ (ppm) = 7.91-7.74 (m, 6 H, C_{arom}*H*), 7.54-7.28 (m, 9 H,C_{arom}*H*), 7.23 (t, 1 H, $^3J_{H,H}$ = 6.0 Hz, CH_2N*H*CO), 6.63 (d,1 H, $^3J_{H,H}$ = 8.6 Hz, CHN*H*CO), 6.10 (t, 1 H,$^3J_{H,H}$ = 5.6 Hz, CON*H*CH_2), 4.42-4.31 (m, 1 H, NHC*H*CO), 3.64-3.52(m, 2 H, CH_2NHCO), 3.61 (d, 1 H,$^3J_{H,H}$ = 1.9 Hz, C*H*CH), 3.58 (d, 1 H,$^3J_{H,H}$ = 1.9 Hz, CHC*H*), 3.34-3.17 (m, 2H, NHC$H_2$$CH_2$), 2.32 (dt, 2 H,$^3J_{H,H}$ = 6.8 Hz, $^4J_{P,H}$ = 3.1 Hz, C_{Cp}C$H_2$$CH_2$), 1.69-1.48 (m, 4 H, CHCH_2CH, CHCH_2C*H*$(CH_3)_2$, CH_2CH_2C*H*$(CH_3)_2$), 1.58 (d, 3 H,$^4J_{P,H}$ = 2.4 Hz, C_{Cp}CH_3), 1.56 (d, 3 H,$^4J_{P,H}$ = 2.3 Hz, C_{Cp}CH_3), 1.39 (q, 2 H,$^3J_{H,H}$= 7.8 Hz, CH_2CH_2CH$(CH_3)_2$), 1.11 (d, 3 H,$^4J_{P,H}$ = 3.4 Hz, C_{Cp}CH_3), 1.10 (d, 3 H$^4J_{P,H}$ = 3.0 Hz, C_{Cp}CH_3), 0.92 (d, 6 H$^3J_{H,H}$ = 6.6 Hz, CH(CH_3)$_2$), 0.88 (d, 6 H,$^3J_{H,H}$ = 6.1 Hz, CH(CH_3)$_2$).

^{31}P-NMR (162 MHz, $CDCl_3$, RT): δ (ppm) = 29.2 (d, $^1J_{Rh,}$P = 142.2 Hz);

ESI-MS: m/z [%] = 860.1 $[M\text{-}Cl]^+$ (100).

1.3.4 Ruthenium-conjugated affinity label $\mathbf{2^{Ru}}$

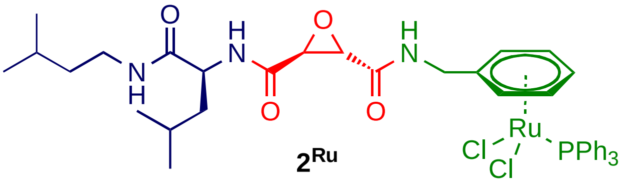

2$^{\mathbf{Ru}}$

$C_{40}H_{48}Cl_2N_3O_4PRu$ (823.75): calcd. C 57.35, H 5.77, N 5.02; found C 57.47, H 5.29, N 4.71.

Product: orange-red solid (80 mg, 0.095 mmol 95%)

^{1}H-NMR (400 MHz, $CDCl_3$, RT): δ (ppm) = 8.04 (t, 1 H, $^3J_{H,H}$ = 5.8 Hz, $CH_2N\mathit{H}CO$), 7.75-7.65 (m, 6 H, $C_{arom}\mathit{H}$), 7.48-7.36 (m, 9 H, $C_{arom}\mathit{H}$), 7.06 (d, 1 H, $^3J_{H,H}$ = 8.5 Hz, CON*H*CH), 6.50 (t, 1 H, $^3J_{H,H}$ = 5.6 Hz, CON*H*CH_2), 5.65 (d, 1 H, $^3J_{H,H}$ = 5.6 Hz, $C_{arom}\mathit{H}$), 5.39 (m, 1 H, $C_{arom}\mathit{H}$), 5.33 (d, 1 H, $^3J_{H,H}$ = 5.5 Hz, $C_{arom}\mathit{H}$), 5.16 (m, 1 H, $C_{arom}\mathit{H}$), 4.67-4.58 (m, 2 H, $C_{arom}\mathit{H}$, NHC*H*CO), 4.49-4.34 (m, 2 H, $C_{arom}C\mathit{H_2}NH$), 3.92 (s, 1 H, C*H*CH), 3.64 (s, 1 H, CHC*H*), 3.33-3.13 (m, 2 H, $NHC\mathit{H_2}CH_2$), 1.69-1.52 (m, 4 H, $CHC\mathit{H_2}CH$, $CHCH_2C\mathit{H}(CH_3)_2$, $CH_2CH_2C\mathit{H(CH_3)_2}$), 1.39 (q, 6 H, $^3J_{H,H}$ = 7.3 Hz, $CH_2CH_2C\mathit{H(CH_3)_2}$), 0.91-0.87 (m, 12 H, $CH(C\mathit{H_3})_2$).

^{31}P-NMR (162 MHz, $CDCl_3$, RT): δ (ppm) = 27.3 (s);

ESI-MS: m/z [%] = 802.2 [M-Cl]+ (100).

1.3.5 Rhodium-conjugated non-covalent metalla affinity label $\mathbf{3^{Rh}}$

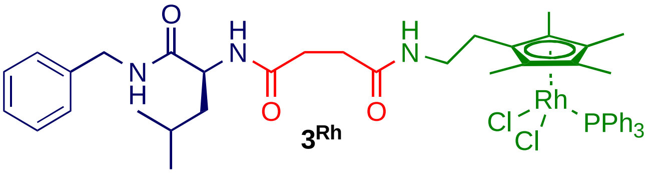

3$^{\mathbf{Rh}}$

Product: red solid (54 mg, 0.059 mmol, 73 %).

$C_{46}H_{55}N_3O_4PRh$ (902.73): calcd. C 61.20, H 6.14, N 4.65; found C 60.57, H 5.76, N 4.42.

^{1}H-NMR (400 MHz, $CDCl_3$, RT): δ (ppm) = 7.82-7.74 (m, 6H, $C_{PPh3}\mathit{H}$), 7.48-7.11 (m, 16H, $N\mathit{H}CH_2$ / $C_{PPh3}\mathit{H}$ / $C_{Bn}\mathit{H}/N\mathit{H}CH_2$), 6.46 (d, 1H, $^3J_{HH}$ = 8.1 Hz, N*H*CH), 4.49-4.40 (m, 1H, NHC*H*), 4.36 (d, 2H, $^3J_{HH}$ = 5.9 Hz, $NHC\mathit{H_2}Ph$), 3.52-3.33 (m, 2H, $CH_2C\mathit{H_2}NH$), 2.60-2.38 (m, 4H, $COC\mathit{H_2}C\mathit{H_2}CO$), 2.30-2.20 (m, 2H, $C\mathit{H_2}CH_2NH$), 1.83-1.50 (m, 3H, $C\mathit{H_2}C\mathit{H}(CH_3)_2$), 1.49 (d, 3H, $^4J_{PH}$ = 2.8 Hz, $C_{Cp}C\mathit{H_3}$), 1.48 (d, 3H, $^4J_{PH}$ = 2.5 Hz, $C_{Cp}C\mathit{H_3}$), 1.03 (d, 3H, $^4J_{PH}$ = 3.3 Hz, $C_{Cp}C\mathit{H_3}$), 0.99 (d, 3H, $^4J_{PH}$ = 2.9 Hz, $C_{Cp}C\mathit{H_3}$), 0.89 (d, $^3J_{HH}$ = 4.6 Hz, $CHC\mathit{H_3}$), 0.87 (d, $^3J_{HH}$ = 4.6 Hz, $CHC\mathit{H'_3}$).

^{13}C-NMR (101 MHz, $CDCl_3$, RT): δ (ppm) = 172.9 / 172.7 / 172.4 (each s, *C*ON), 138.9 (s, C_{Bn}), 134.8 (d, $^3J_{PC}$ = 9.5 Hz, C_{PPh3}), 130.7 (br s, C_{PPh3}), 128.6 (s, C_{Bn}H), 128.2 (br s, C_{PPh3}), 127.7 (s, C_{Bn}H), 127.2 (s, C_{Bn}H), 104.0 (d, $^1J_{RhC}$ = 6.0 Hz, C_{Cp}), 102.9 (d, $^2J_{RhC}$ = 6.0 Hz, C_{Cp}), 101.0-101.3 (m, C_{Cp}), 96.0 (d, $^2J_{RhC}$ = 6.9 Hz,C_{Cp}), 95.4 (d, $^1J_{RhC}$ = 7.6 Hz,C_{Cp}), 52.2 (s, $\mathit{C}HCH_2$), 43.4 (s, $NH\mathit{C}H_2$), 40.7 (s, $CH\mathit{C}H_2$), 36.1 (d, $^4J_{PC}$ = 4.7 Hz, $\mathit{C}H_2CH_2NH$), 32.1 / 31.7 (each s, $\mathit{C}H_2C'H_2$ / $CH_2\mathit{C}'H_2$), 25.0 (s, $\mathit{C}H(CH_3)_2$), 24.7 (d, $^4J_{PC}$ = 2.3 Hz, $CH_2\mathit{C}H_2NH$), 23.2 / 21.9 (each s, $\mathit{C}H_3$ / $\mathit{C}'H_3$), 9.7 / 9.6 / 8.7 / 8.6 (each s, $C_{Cp}\mathit{C}H_3$).

^{31}P-NMR (162 MHz, $CDCl_3$, RT): δ (ppm) = 29.8 (d, J_{PRh} = 142.0 Hz).

ESI-MS m/z [%] = 604.2 $[M\text{-}PPh_3\text{-}Cl]^+$ (100), 866.0 $[M\text{-}Cl]^+$ (20).

2 Conjugation of m-ALs to cysteine proteases

2.1 Preparation of organometallic enzyme hybrids for MALDI-TOF/TOF analysis

2.1.1 MALDI TOF/TOF analysis of papain based organometallic enzyme hybrids: 50 μL of a papain suspension were dissolved in 2950 μL of phosphate buffer (40 mM, pH 7.0), containing 1 mM DTT. To 50 μL of this solution 5 μL of metalla-affinity label (0.342 mM in DMSO) were added and incubated for 2 h at RT. Prior to measurement, the samples were filtered using Millipore ZipTips C4 to remove buffer salts. The samples were analyzed with a Bruker Ultraflex TOF/TOF with a SOUT-MTP ion source, samples were prepared in α-cyanohydroxycinnamic acid, laser pulse at 337 nm, ~100 mJ, 1 ns pulse width. Spectra were calibrated to the residual papain peak.

2.1.2 MALDI TOF/TOF analysis of Cathepsin L based organometallic enzyme hybrids: Cathepsin L is diluted to a final concentration of 0.3 mg/ml in 20 mM sodium acetate at pH = 5.0 containing 100 mM sodium chloride and 1 mM DTT. To 50 μl of the enzyme solution, 5 μl affinity label in DMSO (0.342 mM) are added and incubated for 2 hours at RT The samples are prepared using Millipore ZipTips C4 to remove buffer salts.

Table S-1. Detected most abundant monoionic species in MALDI-TOF/TOF analysis of organometallic enzyme hybrids.

OMEH		Mass [Da]		assignment	
enzyme	m-AL	observed	$\Delta m_{OMEH\text{-}protease}$	Composition	Δm expected
papain	-	23394	-	$[papain]^+$	-
papain	**1**	23726	332	[papain+**1**]$^+$	334
papain	**2**	23713	319	[papain+**2**]$^+$	313
papain	$\mathbf{1^{Rh}}$	23976	582	[papain+$\mathbf{1^{Rh}}$-2Cl-PPh_3]$^+$	583
papain	$\mathbf{1^{Ru}}$	23814	420	[papain+$\mathbf{1^{Ru}}$-2Cl-PPh_3-Ru]$^+$	423
papain	$\mathbf{2^{Rh}}$	23961	567	[papain+$\mathbf{2^{Rh}}$-2Cl-PPh_3]$^+$	563
papain	$\mathbf{2^{Ru}}$	23795	401	[papain+$\mathbf{2^{Ru}}$-2Cl-PPh_3-Ru]$^+$	403
papain	$\mathbf{3^{Rh}}$	23396	2	$[papain]^+$	-
cathepsin L	**-**	24117 / 24240 / 24385 a)	--	[cathepsin L]$^+$	-
cathepsin L	**1**	24446 / 24574 / 24724 a)	334 b)	[cathepsin L+**1**]$^+$	334
cathepsin L	**2**	24423 / 24546 / 24694 a)	308 b)	[cathepsin L+**2**]$^+$	313
cathepsin L	$\mathbf{1^{Rh}}$	24704 / 24828 / 24969 a)	586 b)	[catL+$\mathbf{1^{Rh}}$-2Cl-PPh_3]$^+$	583
cathepsin L	$\mathbf{1^{Ru}}$	24537 / 24669 / 24810 a)	424 b)	[catL+$\mathbf{1^{Ru}}$-2Cl-PPh_3-Ru]$^+$	423
cathepsin L	$\mathbf{2^{Rh}}$	24666 / 24796 / 24943 a)	554 b)	[catL+$\mathbf{2^{Rh}}$-2Cl-PPh3]$^+$	563
cathepsin L	$\mathbf{2^{Ru}}$	24534 / 24638 / 24776 a)	410 b)	[catL+$\mathbf{2^{Ru}}$-2Cl-PPh_3-Ru]$^+$	403

a) the cathepsine L sample showed 3 mass peaks with approximate difference of 123 Da and 145 Da. This pattern was observed independently of the MALDI-matrix applied. b) Δm=average of mass difference for all three peaks.

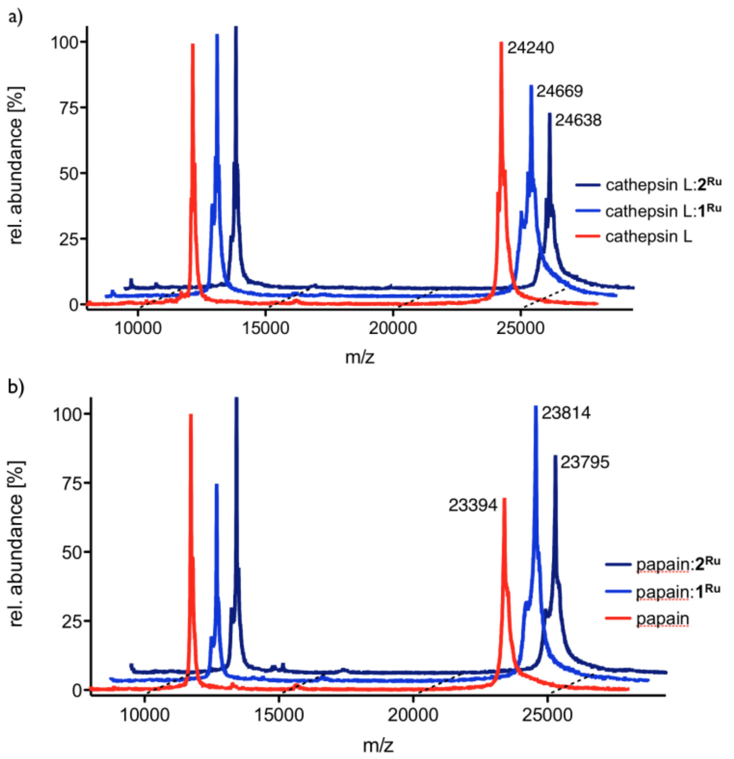

Figure S-1. a) MALDI-TOF mass spectra of cathepsin L before and after incubation with m-ALs $\mathbf{1}^{\mathbf{Ru}}$ and $\mathbf{2}^{\mathbf{Ru}}$; b) MALDI-TOF mass spectra of papain before and after incubation with m-ALs $\mathbf{1}^{\mathbf{Ru}}$ and $\mathbf{2}^{\mathbf{Ru}}$.

2.2 Chromogenic assays

The Papain suspension was diluted 1/100 (final conc. approx. 0.22 mg/ml) in 40 mM phosphate buffer (pH = 7.0), containing 2 mM DTT. Subsequently, to 200 µl of the prepared papain solution the appropriate amount of a DMSO solution of the respective label (0-7 µL, 0.34mM) was added, followed by DMSO (7 - 0 µL) to achieve a constant concentration of the organic solvent in all samples. After 45 min incubation time (3 h for m-AL **3**$^{\mathbf{Rh}}$), BAPNA (20 µl, 1 mM in DMSO/40 mM phosphate buffer = 1/1) was added to each sample (total volume 227 µl, c_{papain}= 8.3µM; c_{label}= 0 – 10.5 µM) and the measurement was started on a Cary 50 UV-Vis spectrometer. Absorbance was measured at 410 nm for 125 min at 25 °C.

1@papain[a)]

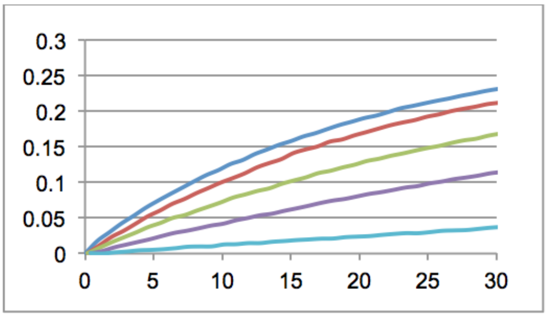

2@papain[b)]

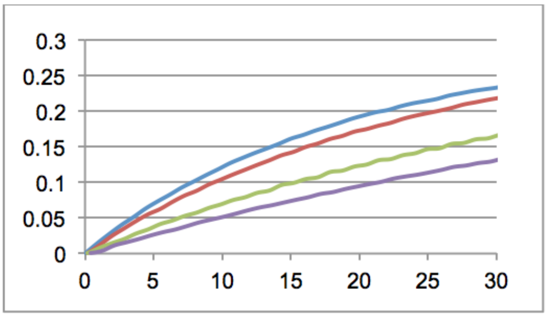

1$^{\mathbf{Rh}}$@papain[c)]

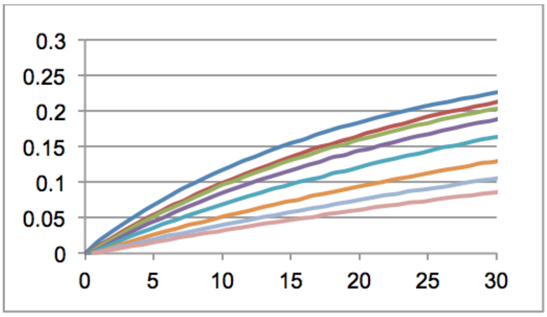

2$^{\mathbf{Rh}}$@papain[c)]

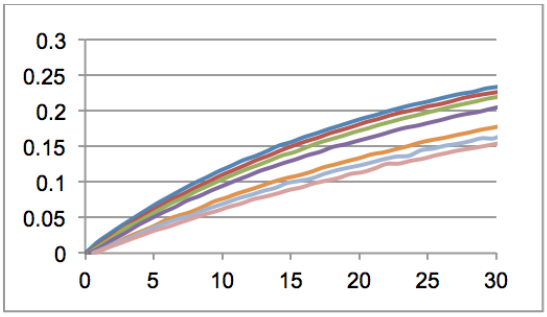

1$^{\mathbf{Ru}}$@papain[c)]

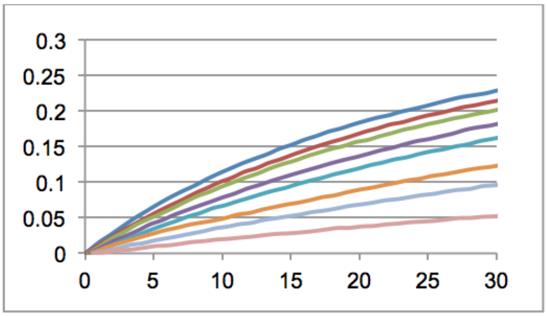

2$^{\mathbf{Ru}}$@papain[c)]

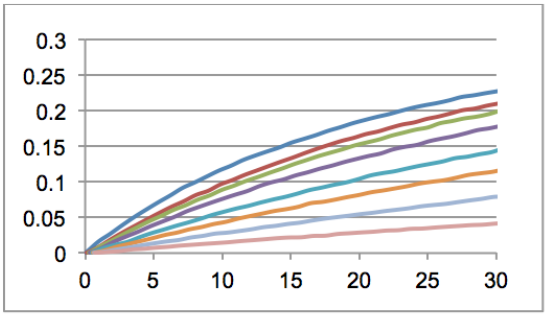

Figure S-2. Remaining proteolytic activity of papain incubated with different m-ALs. x-axis: time in minutes y-axis: absorption. The following concentrations of m-AL were added: a) 0.00 mM / 1.58 mM / 3.16 mM / 6.32 mM / 9.48 mM; b) 0.00 mM / 1.58 mM / 3.16 mM / 6.32 mM; c) 0.00 mM / 1.58 mM / 3.16 mM / 4.74 mM / 6.32 mM / 7.90 mM / 9.48 mM / 11.06 mM.

Comparison of m-AL **1**^{Rh} and **3**^{Rh}. The Papain suspension was diluted 1/100 (final conc. approx. 0.22 mg/ml) in 40 mM phosphate buffer (pH = 7.0), containing 2 mM DTT. Subsequently, to 200 μl of the prepared papain solution 5 μL of the respective m-AL solution (0.0 μM, 4.7 μM, 24, 95 μM, 120 μM, 140 μM, 190 μM, 240 μM, 300 μM, 400μM in DMSO) were added. After 3 h incubation time BAPNA (10 μL, 1 mM in DMSO / 40 mM phosphate buffer = 1/1) was added to each sample (total volume 215 μL, c_{papain}= 8.7 μM; c_{label}= 0 – 9.5 μM) and the measurement was started on a Genios 96-well plate reader. Absorbance was measured at 410 nm for 250 min at 25 °C.

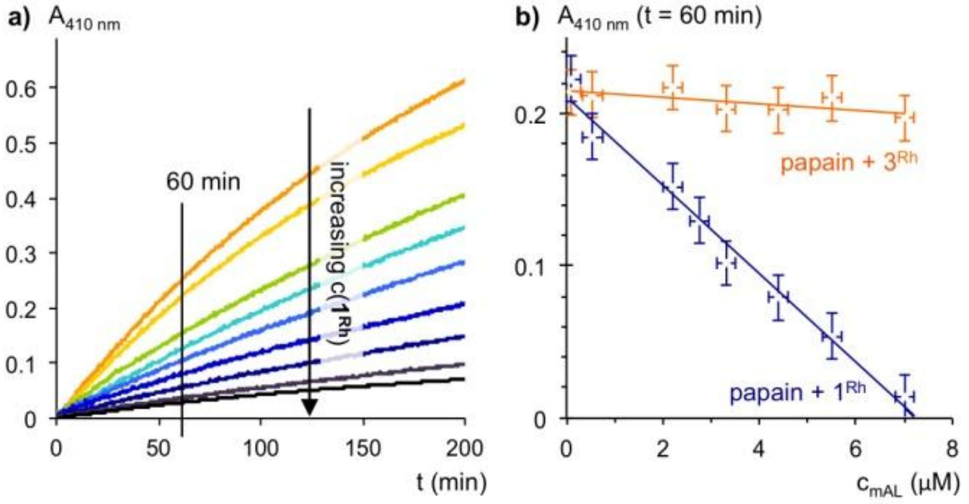

Figure S-3. (a) Chromogenic assay monitoring papain's activity for proteolytic cleavage of BAPNA at different concentrations of m-AL **1**^{Rh} over 200 min. (b) Absorption measured at t = 60 min; papain inhibition by **1**^{Rh} (blue) or **3**^{Rh} (orange).

3 Hydrogenation of ketones using organometallic enzyme hybrids

3.1 General procedure for hydrogenation of ketones

General procedure for asymmetric hydrogenation of ketones catalyzed by organometallic enzyme hybrids: A 1 mL glass vial, equipped with a stirring bar was charged with 200 μL of protease suspension (c = 22 mg/mL) and 260 μL aqueous buffered solution (120 mM PO_4^{2-}) containing DTT (0 to 7.7 mM). After incubating for 1 h, 50 μL DMSO and 5 μL of the m-AL (30 mM in DMSO) were added and the solution was stirred gently for 3 h. Subsequently 5μL **1-OH** (20 mM in DMSO) were added and the reaction solution was stirred for another 3 h, before 5 μL of ketone (2 M in DMSO) was added. The vial was placed in a standard multivial autoclave and pressurized with H_2 (10 to 75 bar) at the desired temperature (20 to 40 °C). After the desired amount of time, pressure was released and the reaction solution was extracted with dichloromethane (600 μL, 2.5 h). Yields were determined either with ^{19}F NMR spectroscopy or gas chromatography. Enantiomer enrichment was determined by chiral GC-FID (Chirasil-Dex).

Substoichometric addition of the catalyst motive ensures that m-ALs bind to the protease and that metal centers are embedded in a chiral environment. However, after addition and binding of the m-AL, residual protease activity can be detected. If quenching of the residual protease activity by addition of **1-OH** is neglected, self-cleavage of the protease results in slow digestion of the OMEH catalyst leading to irreproducible results.

Overall, efficient covalent anchoring of the m-AL in the protease's binding site is a key-factor to achieve good catalytic activities and stereoinduction. This was also reflected in a pronounced dependence of the OMEH catalyst's performance on the composition of the protease batch used, which we encountered during our studies. Hence, it is mandatory to control the quality of the enzyme batches used. We realized that the quality of commercially available papain and bromelain varies from batch to batch and even more from vendor to vendor. Low quality batches lead to reduced yields and lower enantiomer enrichment. Usually, batches of lower quality can already be identified by a substantial amount of impurities visible by the MALDI MS. To ensure comparability of the results presented in this study, all reactions were conducted with enzymes of the highest quality level (twice recrystallized) available from Sigma Aldrich. For any new batch we recorded a MALDI-MS spectrum of the protease and tested its proteolytic activity using the chromogenic assay described above. Further a test run with m-AL $\mathbf{1^{Rh}}$ and substrate S-4 was carried out under standard conditions (see below) to identify potential changes in performance. This way we achieved good reproducibility of our results.

3.2 Prochiral substrates for asymmetric hydrogenation used in this work

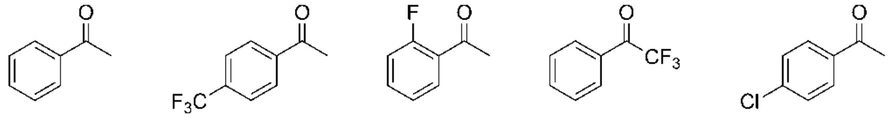

S-1 **S-2** **S-3** **S-4** **S-5**

Figure S-3. Hydrogenation substrates.

3.3 Variation of different parameters

If not mentioned otherwise, standard reaction conditions for hydrogenations are:
120 mM phosphate buffer, pH = 6.5, p(H_2) = 25 bar, t = 65 h, protease host: papain, c(protease) ca. 0.36 mM, c(m-AL) = 0.28 mM, c(substrate) = 20 mM, DMSO content: 12.4 %, c(DTT) = 0.95 mM, substrate **S-4**.

Table S-2. Variation of DMSO concentration (reaction conditions: T = 30 °C, 1.9 mM DTT).

enzyme	m-AL	DMSO / [%]	yield / [%]	% major
papain	$\mathbf{1^{Rh}}$	12.4	81[a)]	60 *(R)*
papain	$\mathbf{1^{Rh}}$	12.4	16	62 *(R)*
papain	$\mathbf{1^{Rh}}$	8.0	4	61 (*R*)
papain	$\mathbf{1^{Rh}}$	5.2	3	58 (*R*)
papain	$\mathbf{1^{Rh}}$	3.2	< 1	n.d.

[a)] T = 40 °C, 0.95 mM DTT

Table S-3. Variation of reaction temperature (reaction conditions: 3.8mM DTT).

enzyme	m-AL	*T* / [°C]	yield / [%]	% major
papain	$\mathbf{1^{Rh}}$	20	10	62 (*R*)
papain	$\mathbf{1^{Rh}}$	30	16	62 (*R*)
papain	$\mathbf{1^{Rh}}$	40	53	62 (*R*)
papain	$\mathbf{3^{Rh}}$	20	2	53 (*R*)
papain	$\mathbf{3^{Rh}}$	40	14	55 (*R*)

Table S-4. Variation of hydrogen pressure (reaction conditions: T = 40 °C, 0.95mM DTT).

enzyme	m-Al	$p(H_2)$ / [bar]	yield/ [%]	% major
papain	$\mathbf{1^{Rh}}$	10	10	56 (*R*)
papain	$\mathbf{1^{Rh}}$	25	81	62 (*R*)
papain	$\mathbf{1^{Rh}}$	75	89	60 (*R*)
papain	$\mathbf{3^{Rh}}$	10	6	52 (*R*)
papain	$\mathbf{3^{Rh}}$	25	26	53 (*R*)
papain	$\mathbf{3^{Rh}}$	75	21	52 (*R*)

Table S-5. Variation of pH value (reaction conditions: T = 40 °C, 0.95mM DTT).

enzyme	m-AL	pH	yield / [%]	% major
papain	$\mathbf{1^{Rh}}$	6.0	33	60 (*R*)
papain	$\mathbf{1^{Rh}}$	6.5	81	62 (*R*)
papain	$\mathbf{1^{Rh}}$	7.0	39	60 (*R*)
papain	$\mathbf{1^{Rh}}$	7.5	42	58 (*R*)
papain	$\mathbf{3^{Rh}}$	6.0	7	< 53
papain	$\mathbf{3^{Rh}}$	6.5	26	54 (*R*)
papain	$\mathbf{3^{Rh}}$	7.0	37	54 (*R*)
papain	$\mathbf{3^{Rh}}$	7.5	33	53 (*R*)

Table S-6. Variation of DTT concentration (reaction conditions: T = 40 °C, pH = 6.5).

enzyme	m-AL	DTT / mM	yield / [%]	% major
papain	$\mathbf{1^{Rh}}$	3.8	53	62 (*R*)
papain	$\mathbf{1^{Rh}}$	2.9	57	61 (*R*)
papain	$\mathbf{1^{Rh}}$	1.9	58	61 (*R*)
papain	$\mathbf{1^{Rh}}$	0.95	81	62 (*R*)
papain	$\mathbf{1^{Rh}}$	0.48	36	58 (*R*)
papain	$\mathbf{1^{Rh}}$	0.19	19	51 (*R*)
papain	$\mathbf{1^{Rh}}$	0.01	21	51 (*R*)
papain	$\mathbf{1^{Rh}}$	0	49	54 (*S*)
papain	$\mathbf{3^{Rh}}$	3.8	14	55 (*R*)
papain	$\mathbf{3^{Rh}}$	2.9	20	53 (*R*)
papain	$\mathbf{3^{Rh}}$	1.9	15	52 (*S*)
papain	$\mathbf{3^{Rh}}$	0.95	27	51 (*S*)
papain	$\mathbf{3^{Rh}}$	0	30	52 (*S*)

Table S-7. Hydrogenations with $\mathbf{1^{Ru}}$.

enzyme	m-AL	$p(H_2)$ / bar	*t* / [h]	yield / [%]	% major
papain	$\mathbf{1^{Ru}}$	75	96	44	60 (*S*)
papain	$\mathbf{1^{Ru}}$	25	65	19	63 (*S*)
bromelain	$\mathbf{1^{Ru}}$	75	96	44	60 (*S*)
bromelain	$\mathbf{1^{Ru}}$	25	65	18	63 (*S*)
-	$\mathbf{1^{Ru}}$	25	65	5	< 53

Table S-8. Hydrogenations with bromelain as host protein**.**

enzyme	m-Al	T / °C	pH	$p(H_2)$ / bar	DMSO / [%]	DTT [mM]	t / [h]	yield / [%]	% major
bromelain	$\mathbf{1^{Rh}}$	40	6.5	75	12.4	3.8	96	36	< 51
bromelain	$\mathbf{1^{Rh}}$	20	6.5	25	12.4	3.8	65	3	< 53
bromelain	$\mathbf{1^{Rh}}$	40	6.5	25	12.4	0.95	65	11	56 (*S*)
bromelain	$\mathbf{1^{Ru}}$	40	6.5	75	12.4	0.95	96	44	60 (*S*)
bromelain	$\mathbf{1^{Ru}}$	40	6.5	25	12.4	0.95	65	18	63 (*S*)
bromelain	$\mathbf{2^{Rh}}$	40	6.5	25	12.4	0.95	65	17	54 (*S*)
bromelain	$\mathbf{2^{Rh}}$	40	6.5	75	12.4	0.95	96	36	57 (*S*)

Table S-9. Hydrogenations with $\mathbf{2^{Rh}}$, $\mathbf{3^{Rh}}$ or without host protein**.**

enzyme	m-Al	T [°C]	pH	p (H_2) [bar]	DTT [mM]	t [h]	yield [%]	% major
papain	$\mathbf{2^{Rh}}$	40	6.5	25	0.95	65	41	60 (*R*)
bromelain	$\mathbf{2^{Rh}}$	40	6.5	25	0.95	65	36	57 (*S*)
-	$\mathbf{2^{Rh}}$	20	6.5	25	3.8	65	<1	n.d.
-	$\mathbf{2^{Rh}}$	40	6.5	25	0	65	4	< 51
papain	$\mathbf{3^{Rh}}$	40	6.5	25	0.95	65	26	53 (*R*)
-	$\mathbf{3^{Rh}}$	40	6.5	25	0.95	65	6	< 51
-	$\mathbf{3^{Rh}}$	40	6.5	25	0	65	4	52 (*R*)
-	$\mathbf{1^{Rh}}$	40	6.5	25	0.95	65	7	51 (*S*)
-	$\mathbf{1^{Rh}}$	20	6.5	25	3.8	65	<1	n.d.
-	$\mathbf{1^{Rh}}$	40	6.5	25	0	65	4	51 (*R*)
-	$\mathbf{1^{Ru}}$	40	6.5	25	0.95	65	6	< 51

4 References

[1] W.H. Ang, E. Daldini, L. Juillerat-Jeanneret, P.J. Dyson, *Inorg. Chem.* **2007**, *46*, 9048-9050.

[2] T. Reiner, M. Waibel, A. N. Marziale, D. Jantke, F. J. Kiefer, T. F. Fässler, J. Eppinger, *J. Organomet. Chem.* **2010**, *695*, 2667-2672.

[3] D. van Leusen, D. J. Beetstra, B. Hessen, J. H. Teuben, *Organometallics* **2000**, *19*, 4084-4089.

[4] T. Reiner, D. Jantke, A. Raba, A. N. Marziale, *J. Organomet. Chem.* **2009**, *694*, 1934-1937.

[5] (a) B. J. Gour-Salin, P. Lachance, M.-C. Magny, C. Plouffe, R. Ménard, A. C. Storer, *Biochem. J.* **1994**, *299*, 389-392; (b) K. A. H. Chehade, A. Baruch, S. H. L. Verhelst, M. Bogyo, *Synthesis* **2005**, *2*, 240-244.
